# Clinician Trends in Prescribing Direct Oral Anticoagulants for US Medicare Beneficiaries

**DOI:** 10.1001/jamanetworkopen.2021.37288

**Published:** 2021-12-06

**Authors:** Kevin M. Wheelock, Joseph S. Ross, Karthik Murugiah, Zhenqiu Lin, Harlan M. Krumholz, Rohan Khera

**Affiliations:** 1 Department of Internal Medicine, Yale School of Medicine, New Haven, Connecticut; 2 Center for Outcomes Research and Evaluation, Yale-New Haven Hospital, New Haven, Connecticut; 3 Section of General Medicine, Department of Internal Medicine, Yale School of Medicine, New Haven, Connecticut; 4 Department of Health Policy and Management, Yale School of Public Health, New Haven, Connecticut; 5 Section of Cardiovascular Medicine, Department of Internal Medicine, Yale School of Medicine, New Haven, Connecticut

## Abstract

**Importance:**

Contemporary national clinical practice guidelines recommend direct-acting oral anticoagulants (DOACs) as the first-line anticoagulant strategy over warfarin for most indications, especially among older individuals with an elevated bleeding risk.

**Objective:**

To evaluate anticoagulant prescribing and DOAC uptake by US clinicians in the Medicare population.

**Design, Setting, and Participants:**

This retrospective cohort study included all US clinicians with more than 10 Medicare oral anticoagulant prescription claims, who were included in the national Medicare Provider Utilization and Payment Data (2013-2018). Data analyses were conducted between October 2020 and October 2021.

**Exposures:**

DOAC prescription in 2013.

**Main Outcomes and Measures:**

Clinicians were categorized based on 2013 prescribing as solely prescribing warfarin, DOAC, or both, and their temporal trajectories of proportionate DOAC use were examined.

**Results:**

The analysis included 325 666 unique clinicians with more than 10 oral anticoagulant prescriptions between 2013 and 2018 (26 620 [8.2%] cardiologists, 85 563 [26.3%] internal medicine physicians, 84 369 [25.9%] family medicine physicians, and 81 161 [24.9%] advanced practice clinicians, including nurse practitioners and physician assistants). In 2013, among 91 837 prescribers, 54 501 (59.3%) prescribed only warfarin, 1918 (2.1%) prescribed only a DOAC, and 35 418 (38.6%) prescribed both. During the study period, the number of clinicians prescribing DOACs increased, but 19% continued to prescribe only warfarin in 2018. While 359 cardiologists prescribing anticoagulants (1.6%) were warfarin-only prescribers, 10 414 (20.0%) and 6296 (12.6%) of family and internal medicine physicians also prescribed only warfarin, respectively. Clinicians prescribing only warfarin in 2013 had lower proportionate DOAC use throughout the study compared with 2013 DOAC prescribers, which represents a median (IQR) of 41.9% (20.3%-61.9%) of their anticoagulant prescriptions in 2018 vs 67.0% (49.9%-82.8%) for DOAC prescribers.

**Conclusions and Relevance:**

Despite the increase in DOAC use among Medicare beneficiaries, many clinicians in this study continued to use warfarin as their predominant or only anticoagulant instead of DOACs. There is a need to address barriers to the uptake of these medications to realize their potential benefits for patients.

## Key Points

**Question:**

How have patterns of direct-acting oral anticoagulant (DOAC) use changed among US clinicians between 2013 and 2018?

**Findings:**

In this cohort study of Medicare prescription claim data encompassing 325 666 clinicians from 2013 to 2018, most clinicians continued to use warfarin as their predominant or only anticoagulant instead of DOACs, including 1 in 5 general medicine practitioners exclusively using warfarin in 2018. Despite an increase in DOAC prescribing, those prescribing only warfarin in 2013 had lower proportionate DOAC use throughout the study than 2013 DOAC prescribers.

**Meaning:**

In this study, many clinicians did not prescribe any DOACs in 2018, suggesting a need to address barriers to DOAC use.

## Teaser

This cohort study uses Medicare prescription claims data to evaluate patterns of direct oral anticoagulant prescribing by US clinicians between 2013 and 2018.

## Introduction

Warfarin has been the mainstay of oral anticoagulant therapy for nearly 70 years.^1^ However, the last decade has seen the emergence of several direct-acting oral anticoagulants (DOACs) with comparable efficacy for many conditions, significantly lower bleeding risk, and logistically easier use.^2,3,4,5,6,7,8,9^ Therefore, clinical practice guidelines strongly recommend their use over warfarin for many treatment indications. Specifically, in 2019, the American College of Cardiology made a class I recommendation favoring the use of DOACs over warfarin for patients with nonvalvular atrial fibrillation.^10,11^ In 2020, the American Society of Hematology similarly supported the use of DOACs over warfarin to treat venous thromboembolism and pulmonary embolism.^12^ These recommendations are based on randomized trials conducted during the past 15 years, which have shown that DOACs are at least noninferior and, in some cases, superior to warfarin in efficacy with a lower risk of bleeding.^3,4,5,8,9,13,14^ DOACs may be particularly favorable in older individuals who are at an elevated risk of bleeding and are more likely to be challenged by the need for frequent laboratory monitoring.^15^ Despite evidence favoring the use of DOACs over warfarin in many patients, the cost of DOACs remains significantly higher than warfarin, representing a barrier to higher DOAC uptake.^16^

Given the increasing evidence favoring DOACs over warfarin during the past decade and strong, recent recommendations from clinical practice guidelines, it is critical to assess whether clinicians in the US are prescribing these medications and whether their overall patterns of use are consistent with the evidence supporting their role as the primary contemporary oral anticoagulant. Using national part D Medicare prescription data, the present study evaluated whether clinician prescribing practices reflect the changing landscape of evidence favoring DOACs as the primary outpatient oral anticoagulants. Specifically, we assessed DOAC prescribing practices of clinicians across the US between 2013 and 2018, evaluating both the proportion of clinicians prescribing DOACs relative to warfarin and the volume of their prescriptions across part D Medicare beneficiaries over time. Finally, we defined the practice patterns of individual clinicians, identifying patterns in adoption of DOACs over time.

## Methods

This retrospective cohort study followed the Strengthening the Reporting of Observational Studies in Epidemiology (STROBE) reporting guidelines. This study’s data are publicly available, deidentified, and represent non–human subject research under the US Department of Health and Human Services (45 CFR 46) regulations, which precluded informed consent and were outside the purview of the institutional review board at Yale University.

### Data Sources

Using prescriber-level annual national Medicare Provider Utilization and Payment Data from 2013 through 2018, we identified all clinicians (ie, physician assistants and advanced practice clinicians [APCs], which included nurse practitioners and physician assistants) prescribing oral anticoagulants to Medicare beneficiaries. These data encompass Medicare Part D claims processed each year for all beneficiaries in the Part D program, which included 44 249 461 enrollees or 73% of the total Medicare population in 2018.^17^ The data are recorded at the clinician level, and prescriptions for a drug are included if there were 11 or more prescriptions for that drug by a clinician. From these data, we identified the National Provider Identifier number, clinician specialty description, generic drug name, number of beneficiaries prescribed the drug, and the total claim count (ie, original prescriptions and refills).

Furthermore, we used the Doctors and Clinicians National File of the Centers for Medicare & Medicaid Services (CMS) to characterize demographic features of clinicians, which we merged with prescription claims using the National Provider Identifier number. The Doctors and Clinicians National File is updated twice per month with the most current available demographic data on all clinicians who have available data.^18^ From this file, we extracted graduation year for each clinician as a surrogate for clinician experience. To ensure that we included graduation year for as many clinicians as possible, we used the final archived Doctors and Clinicians National File from each year of the study between 2014 and 2018. For 2013, we used the 2014 demographic data, which was the first year this information was publicly reported.

### Study Covariates

We selected commonly prescribed oral anticoagulants; warfarin, dabigatran, rivaroxaban, and apixaban. We also identified prescriptions for 2 additional DOACs, edoxaban and betrixaban. Edoxaban and betrixaban were approved by the US Food and Drug Administration in 2015 and 2017, respectively, and represented 34 370 (0.025%) and 13 (<0.001%) of all DOAC prescriptions. Since their prescription volume was low, the analysis focused on dabigatran, rivaroxaban, and apixaban—the 3 most widely used DOACs.

We also defined key clinician specialties from 171 unique clinician specialties who had prescriptions for an oral anticoagulant. We defined the most common prescriber specialties, which included physicians in cardiology, internal medicine, and family medicine, as well as APCs. Cardiology subspecialties were included under cardiology. Together, these 4 groups accounted for 91.5% of all oral anticoagulant claims across the study period (125 452 368 of 137 116 220 claims). We also examined the prescriptions of hematologists/oncologists, emergency medicine physicians, and vascular surgeons.

### Statistical Analysis

We assessed cumulative oral anticoagulant prescriptions for each year to assess overall trends in prescribing patterns. We stratified these analyses by key clinician specialty groups of cardiology, internal medicine, family medicine, and APCs. Next, we evaluated the number of beneficiaries for a given clinician who received a particular anticoagulant. The exact number of beneficiaries prescribed a drug by a given clinician was reported for counts of 11 and higher, with counts of less than 11 reported as such. In analyses using beneficiaries, we assigned a value of 5 to all clinician-drug combinations, which fell below the threshold. Additionally, we performed a categorical analysis dividing the number of beneficiaries for each clinician-drug combination into 4 categories (<11, 11-20, 21-40, or >40 patients).

Next, we identified individual clinicians for each study year and categorized them into those prescribing warfarin alone, DOACs alone, or both. All clinicians with an oral anticoagulant prescription for any study year were included in this analysis. The difference in prescribing patterns across specialties was assessed using a χ^2^ goodness-of-fit test. For the clinician-level analysis, to explicitly evaluate the experience of clinicians, we stratified trend assessments by graduation year, which was categorized into decades (before 1970, 1971-1980, 1981-1990, 1991-2000, 2001-2010, 2010-present).

Next, we evaluated prescribing patterns of individual clinicians, assessing trends in DOAC prescriptions among those who were early adopters of DOACs. We classified clinicians into categories of warfarin only, DOACs only, or both based on their 2013 oral anticoagulant prescribing practice. These clinicians were then followed over time to assess both their use of DOACs and their volumes of DOAC prescriptions during the study. DOAC prescriptions were modeled as the proportion of all oral anticoagulant prescriptions for each clinician. Clinicians were included in this analysis only if they prescribed an oral anticoagulant prescription in each year of the study.

We then assessed whether changes in DOAC use over time were suggestive of a pattern of increasing use among newer patients or switching existing patients from warfarin to a DOAC. We evaluated this in analyses focusing on temporal changes in prescribing practices of clinicians who prescribed oral anticoagulants to 11 or more beneficiaries in each year of the study. As our exposure, we computed mean annual change in patients receiving anticoagulants for each prescriber using prescriber-level ordinal least squares regression, modeling the number of patients receiving oral anticoagulants over the 6 years of the study. We similarly evaluated the mean annual change in the relative use of DOAC for each prescriber over the same period. We assessed the correlation of these changes over time—a positive correlation indicating that increased DOAC use is explained in part by preferential prescription of DOACs to new patients as opposed to switching existing patients from warfarin to a DOAC.

To evaluate the independent association of baseline DOAC prescribing patterns on subsequent DOAC prescription volume, we constructed a multilevel repeated measures model adjusting for clinician-level baseline prescribing behavior, specialty, graduation year, and time.^19^ Total number of claims and year were modeled as level-1 variables, while baseline prescribing category and clinician graduation year were modeled as level-2 variables. For specialty, cardiology was considered the reference specialty and other specialties (internal medicine, family medicine, and APCs) were considered in relation to cardiology. In a subanalysis, all variables were standardized as units of their SD to permit comparison of the relative effect sizes of each variable across different scales. *P* values were obtained using the Satterwaite degrees of freedom method.

All statistical analyses were conducted in R version 3.6.1 (R Project for Statistical Computing). All *P* values shown are 2-sided, and statistical significance was set at *P*<.05 for all tests. Data analyses were conducted between October 2020 and October 2021.

## Results

There were 325 666 unique clinicians with greater than 10 oral anticoagulant prescriptions between 2013 and 2018 (26 620 [8.2%] cardiologists; 85 563 [26.3%] internal medicine physicians; 84 369 family medicine physicians; and 81 161 [24.9%] APCs) (Table 1). Clinician characteristics were available for 274 295 (84%). After excluding edoxaban and betrixaban, there were 122 645 277 anticoagulant prescriptions by 274 290 clinicians, representing the study population. Internal medicine (76 615 [27.9%]) and family medicine (76 660 [27.9%]) physicians represented the largest proportion of anticoagulation prescribing clinicians. Stratified by year, there were 159 399 clinicians with an oral anticoagulant prescription in 2013, 163 041 in 2014, 161 856 in 2015, 166 041 in 2016, 180 216 in 2017, and 183 964 in 2018.

**Table 1.** Summary Data^a^

Variable	No. (%)	
	Full data set	Complete covariate cohort
Clinicians, No.	325 660	274 290
Prescriptions, No.	137 116 220	122 645 277
Apixaban	20 772 469 (15.1)	19 275 047 (15.7)
Dabigatran	6 344 857 (4.6)	5 886 517 (4.8)
Rivaroxaban	21 035 354 (15.3)	19 416 949 (15.8)
Warfarin	88 963 540 (64.9)	78 066 764 (63.7)
Beneficiaries, No.^b^	2 350 389	2 062 609
<11	1 565 892 (66.6)	1 354 174 (65.7)
11-20	425 592 (18.1)	380 385 (18.4)
21-40	260 309 (11.1)	238 569 (11.6)
>40	98 596 (4.2)	89 481 (4.3)
Specialty		
Cardiology	26 620 (8.2)	26 057 (9.5)
Internal medicine	85 563 (26.3)	76 615 (27.9)
Family medicine	84 369 (25.9)	76 600 (27.9)
Advanced practice clinician	81 161 (24.9)	55 875 (20.4)
Other	47 947 (14.7)	39 143 (14.3)

^a^ Data are shown for the full data set and for clinicians who had complete covariate data (graduation year, used as surrogate for clinician experience) available.

^b^ The number of beneficiaries is shown according to the number of clinician-drug combinations that fall into each beneficiary category.

### Anticoagulant Prescription Volumes in Medicare Part D

**Figure 1.**
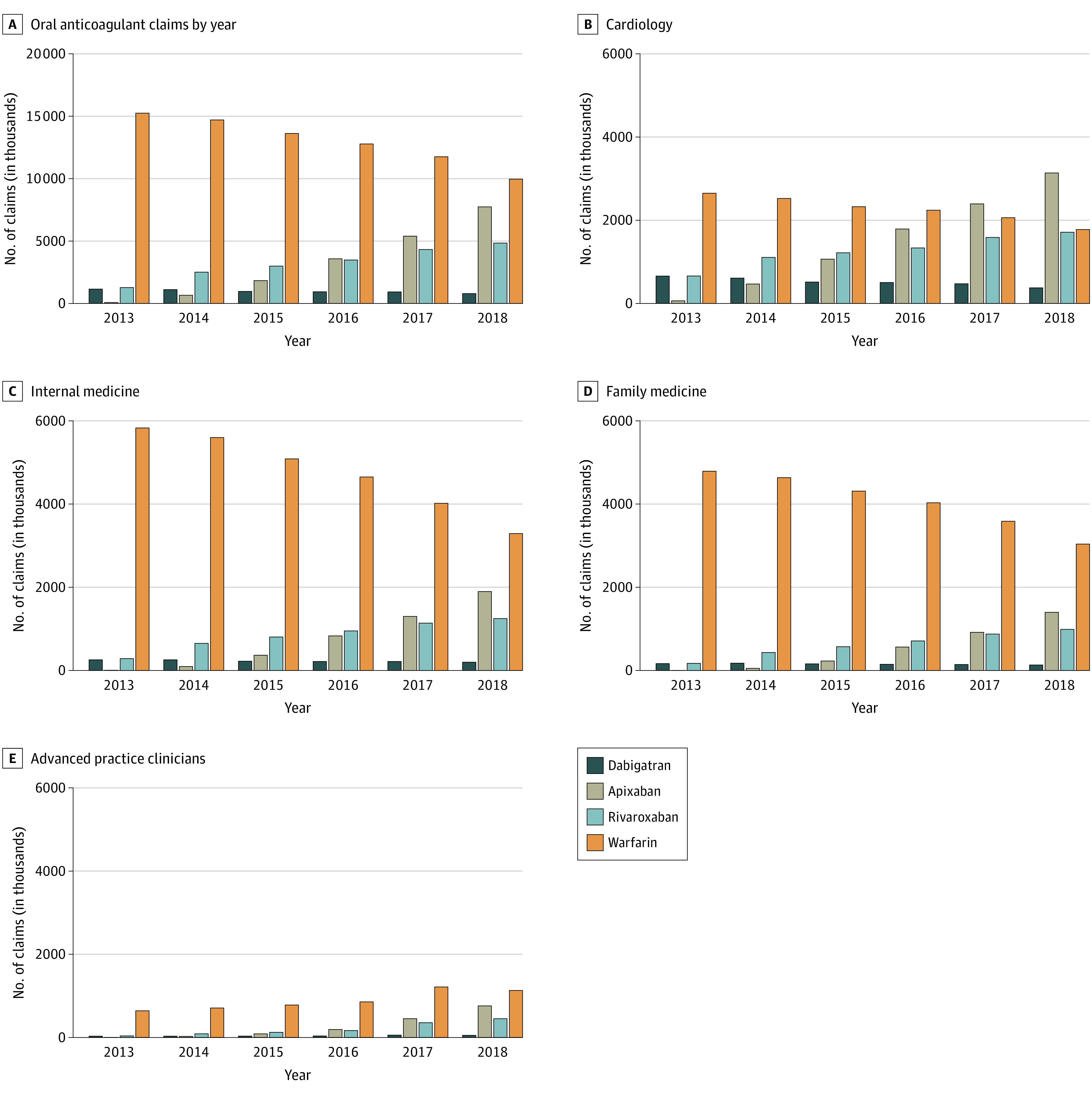
Oral Anticoagulant Claims by Year All available claims for an oral anticoagulant stratified by year. In panel A, all claims included in the study are shown in aggregate. In panels B-E, claims data for cardiologists (B), internal medicine physicians (C), family medicine physicians (D), and advanced practice clinicians (E) are shown.

Throughout this study, both the number of DOAC prescriptions increased as well as DOAC prescriptions as a proportion of all oral anticoagulants (Figure 1). DOAC prescription volume increased from 2 498 712 (14.1% of all anticoagulant prescriptions) in 2013 to 13 368 494 (57.3% of all anticoagulant prescriptions) in 2018, while the warfarin prescription volume decreased from 15 250 397 (85.9% of all anticoagulant prescriptions) in 2013 to 9 959 050 (42.7% of all anticoagulant prescriptions) in 2018. The most frequently used DOACs were apixaban and rivaroxaban, with apixaban increasing from 75 948 to 7 741 247 prescriptions and rivaroxaban increasing from 1 271 758 to 4 835 049 prescriptions between 2013 and 2018. In contrast, prescriptions for dabigatran decreased by 31% (1 151 006 prescriptions in 2013 vs 792 198 prescriptions in 2018). The number of beneficiaries per clinician who prescribed these medications followed the overall prescription volumes (eFigure 1 in the Supplement). Among hematologists/oncologists, vascular surgeons, and emergency medicine clinicians, vascular surgeons had the lowest proportion of warfarin prescriptions in 2018 (11 136 [25.2%]), followed by hematologists/oncologists (155 749 [33.5%]), and emergency medicine physicians (39 556 [48.7%]) (eFigure 2 in the Supplement).

### Warfarin and DOAC Prescribing by US Clinicians

**Figure 2.**
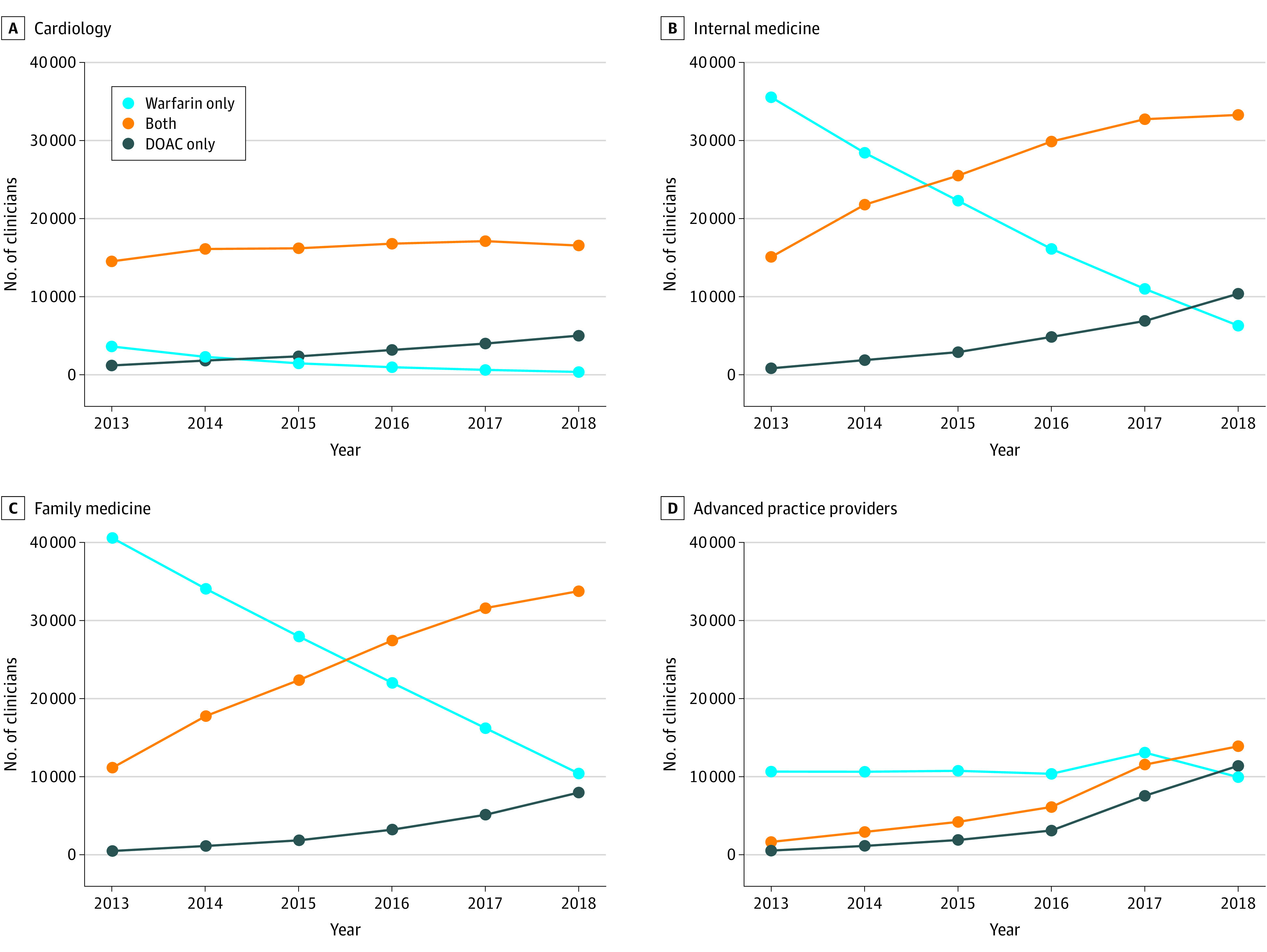
Prescribers in Anticoagulant Category by Year and Specialty Clinicians were divided into prescriber categories each year; individuals who only prescribed warfarin, individuals who prescribed only a direct oral anticoagulant (DOAC), or individuals who prescribed both. For each specialty, the number of clinicians who fell into each category are shown.

There were significant differences in uptake of DOACs across the different specialties (Figure 2). Cardiologists more frequently prescribed a DOAC than internal medicine, family medicine, or APCs. In 2013, 3621 (18.7%) of cardiologists prescribed warfarin as their only oral anticoagulant (ie, prescribed no DOACs) compared with 35 550 (69.1%) of internal medicine physicians, 40 560 (77.7%) of family medicine physicians, and 10 647(83.0%) of APCs (χ^2^ *P* < .001). Among anticoagulant prescribers in 2018, only 359 (1.6%) of cardiologists were not prescribing DOACs. Among the other specialties, there was a large increase in DOAC uptake, but in 2018, 6296 (12.6%) of internal medicine physicians, 10 414 (20.0%) of family medicine physicians, and 9943 (28.2%) of APCs were only prescribing warfarin. Clinicians with more recent graduation years were more likely to prescribe DOACs than clinicians who were further out from graduation (eFigure 3 in the Supplement).

There were large differences between the specialties regarding the number of beneficiaries receiving a given oral anticoagulant from a prescriber (eFigure 4 in the Supplement). Among cardiology clinician-drug combinations during the study, 187 786 (46.1%) were low-volume (<11 beneficiaries for a given drug), compared with 423 948 (66.5%) of internal medicine, 426 867 (71.8%) of family medicine, and 155 923 (74.5%) of APCs.

### Clinician-Level Uptake of DOACs in the US

**Figure 3.**
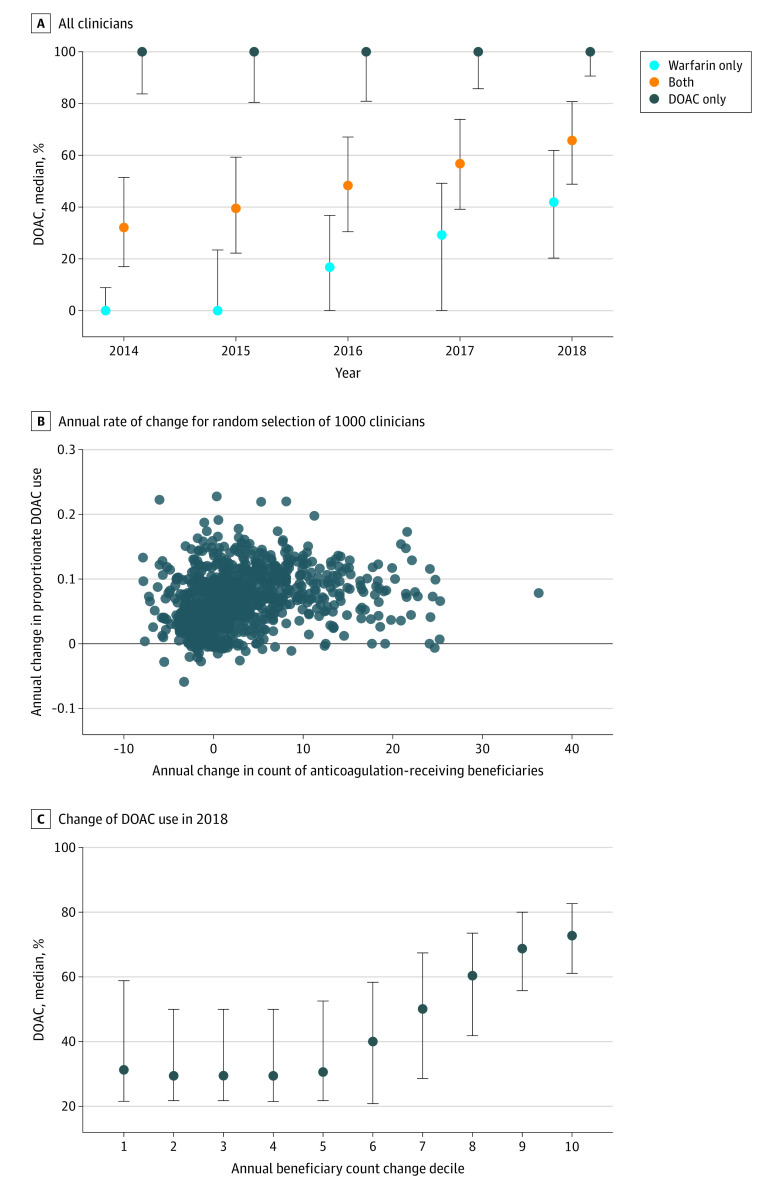
Annual Oral Anticoagulant Prescriber Groups by 2013 Prescribing Behavior In panel A, all clinicians who prescribed an oral anticoagulant from 2013 to 2018 were divided into 3 categories based on 2013 prescriptions: clinicians who only prescribed warfarin, clinicians who prescribed only a DOAC, or clinicians who prescribed both. The proportion of all oral anticoagulants that were DOACs were calculated for each clinician for each subsequent study year. The median (IQR) of the proportion of DOAC scripts for clinicians in each baseline prescriber category are shown. In panel B, the annualized rate of change of DOAC usage and number of beneficiaries is shown for individual clinicians. Panel C shows the 2018 DOAC percentage according to decile of change in beneficiary counts. DOAC indicates direct oral anticoagulants; error bars, interquartile range.

There were 91 837 clinicians who prescribed an oral anticoagulant each year from 2013 to 2018. Of these, 41 713 prescribed to at least 11 beneficiaries. In 2013, 54 501 (59.3%) clinicians prescribed only warfarin, 1918 (2.1%) prescribed only a DOAC, and 35 418 (38.6%) prescribed both. Clinician baseline prescribing practice was associated with their DOAC prescriptions as a proportion of all prescribed oral anticoagulants during the study period. Clinicians who only prescribed warfarin in 2013 had lower relative use of DOACs across the study period, with DOACs representing a median (IQR) of 41.9% (IQR, 20.3%-61.9%) of their anticoagulant prescriptions in 2018 compared with a median (IQR) of 67.0% (IQR, 49.9%-82.8%) among clinicians who prescribed any DOACs (Figure 3A).

Among clinicians who prescribed anticoagulants to at least 11 beneficiaries annually, there was a modest but significant positive correlation between the mean annual change in the number of anticoagulant-receiving beneficiaries and the proportionate use of DOACs (Spearman *r* = 0.31, *P* < .001) (Figure 3B). We also found that clinicians with the largest growth in number of beneficiaries had a greater proportion of DOAC prescriptions in 2018 (median [IQR] DOAC proportion, 0.72 [IQR, 0.61-0.83]) compared with those with the smallest growth in beneficiaries (median [IQR] DOAC proportion, 0.31 [IQR, 0.22-0.59]) (Figure 3C). Additionally, there was a positive correlation between the number of beneficiaries for a given clinician and his or her DOAC use (Spearman *r* = 0.48, *P* < .001). Clinicians who prescribed warfarin only had a median (IQR) of 18 (IQR, 14-26) beneficiaries compared with a median (IQR) of 40 (IQR, 25-72) beneficiaries for those who prescribed DOACs.

In repeated measures models, the factors associated with a higher proportionate use of DOACs were clinician specialty and use of a DOAC in 2013 (Table 2). Compared with cardiologists, family medicine physicians, internal medicine physicians, and APCs prescribed 22.1%, 18.3%, and 19.4% less DOACs, relative to their total oral anticoagulant prescriptions, across the study period. Further, individuals who prescribed any DOACs in 2013 had 21% higher DOAC use across the period compared with those prescribing warfarin.

**Table 2.** Multilevel Model of Proportion of DOAC Prescriptions at the Clinician Level^a^

Variable	Unstandardized		Standardized	
	β estimate (95% CI)	*P* value	β estimate (95% CI)	*P* value
Graduation year	0.001 (0.001 to 0.001)	<.001	0.031 (0.027 to 0.035)	<.001
Clinician specialty				
Internal medicine	−0.183 (−0.187 to −0.180)	<.001	−0.295 (−0.301 to −0.289)	<.001
Family medicine	−0.221 (−0.225 to −0.217)	<.001	−0.356 (−0.362 to −0.349)	<.001
Advanced practice clinician	−0.194 (−0.201 to −0.189)	<.001	−0.146 (−0.151 to −0.141)	<.001
2013 prescriber category				
DOAC only group	0.508 (0.498 to 0.518)	<.001	0.318 (0.313 to 0.322)	<.001
Both DOAC and warfarin group	0.195 (0.192 to 0.198)	<.001	0.217 (0.213 to 0.222)	<.001
Year	0.079 (0.079 to 0.079)	<.001	0.371 (0.369 to 0.372)	<.001

Abbreviation: DOAC, direct-acting oral anticoagulant.

^a^ Clinician graduation year and baseline DOAC prescribing category are level-1 variables, while year and specialty are level-2 variables. The 95% CI is calculated using the Wald test. β estimates are shown as a raw value and standardized to a mean (SD) of 0 (1), allowing for comparison of relative effect sizes.

## Discussion

Between 2013 to 2018, there was a rapid uptake of DOACs among clinician prescribers providing care to Medicare beneficiaries, representing 57.3% of oral anticoagulation prescriptions in 2018. While DOAC prescriptions represented nearly two-thirds of all anticoagulant prescriptions for cardiologists, general medical specialists continued to use warfarin as the preferred anticoagulant in 2018. Moreover, nearly 1 in 5 prescribers in general medical specialties continued to prescribe warfarin as the only anticoagulant to Medicare beneficiaries. Early adoption of DOACs in 2013 was independently associated with both eventual uptake of DOAC use and the proportionate use of DOACs as the anticoagulant of choice, suggesting prescriber inertia reflected in the uptake of DOACs.

Our study represents the largest prescriber-level assessment of national DOAC prescriptions. A previous analysis of Medicare Part D overall prescription volumes between 2013 and 2015 found an increasing use of DOACs, but did not address prescriber practices.^20^ Moreover, these older data may not represent contemporary practices as national clinical practice guidelines increasingly recommend DOACs over warfarin in non-valvular atrial fibrillation and for treatment and prevention of venous thromboembolism.^10,12^

These guideline recommendations are based on evidence that DOACs are both safer and more efficacious compared to warfarin. A meta-analysis was performed on the landmark DOAC trials in 2014, including The Randomized Evaluation of Long-Term Anticoagulation Therapy, or RE-LY trial; Rivaroxaban Once Daily Oral Direct Factor Xa Inhibition Compared with Vitamin K Antagonism for Prevention of Stroke and Embolism Trial in Atrial Fibrillation, or ROCKET AF trial; Apixaban for Reduction in Stroke and Other Thromboembolic Events in Atrial Fibrillation, or ARISTOTLE trial; and Effective Anticoagulation with Factor Xa Next Generation in Atrial Fibrillation—Thrombolysis in Myocardial Infarction 48, or ENGAGE AF-TIMI trial. It found that DOACs reduce the risk of stroke and systemic embolic events by 19% compared with warfarin, while also reducing risk of hemorrhagic stroke by 52%. Notably, their use is associated with a 10% lower all-cause mortality compared with warfarin.^21^ DOACs are particularly beneficial in the elderly population; a meta-analysis of 5 randomized controlled trials comparing DOACs to warfarin found that they were generally more efficacious with lower bleeding risk in patients 75 years and older with nonvalvular atrial fibrillation.^22^ This benefit in elderly patients is particularly important given their higher baseline bleeding risk.

We found that clinicians who were not prescribing DOACs in 2013 remained much less likely to do so throughout the study. This association remained even after accounting for individual prescriber characteristics. Furthermore, we found that prescribers who had a greater increase in the panel of patients receiving anticoagulants over the study period also had a greater increase in DOAC usage, suggesting that the increase in DOAC use was consistent with newer patients seen by a prescriber being prescribed DOACs, rather than transitioning existing patients off warfarin to a DOAC. This pattern suggests both prescriber and patient inertia, and many prescribers likely have patients that could benefit from switching to a DOAC.

There is no defined proportion of patients in a practice that should receive a DOAC, and several patient-level factors guide the choice of oral anticoagulant. However, clinical practice guidelines spanning broad clinical groups suggest DOACs are preferred over warfarin for most patients. Of note, there are key clinical groups where warfarin would be indicated over DOAC, including patients with valvular atrial fibrillation, mechanical heart valve, and antiphospholipid antibody syndrome. Moreover, warfarin would also be preferred over DOACs among patients with end-stage renal disease, Childs-Pugh Class C liver disease, and patients with a left ventricular thrombus.^23,24^ Across large registries of atrial fibrillation and clinical trials evaluating DOACs, fewer than 25% of patients have valvular atrial fibrillation,^3,4,13,25^ and patients with the other conditions represent a minority of all patients who require oral anticoagulation.^26^ Moreover, these patients span clinical domains. Therefore, it is unlikely that a clinician prescribing oral anticoagulants only provides care to patients with warfarin-specific medical indications or patients who are unable to afford a DOAC. This pattern suggests prescriber and patient inertia, and many prescribers may have patients that could benefit from switching to a DOAC.^27^

In clinical practice, the cost of DOACs may represent a barrier to more widespread adoption. However, even at current costs, at a population level, there is evidence that DOACs are cost-effective compared with warfarin because of lower thrombotic events, bleeding risk, and the lack of need for drug monitoring.^28,29^ Moreover, an analysis of Medicare Prescription Drug Plan formularies suggested that all formularies offered DOAC coverage in 2017,^16^ though only 32% offered unrestricted coverage, with most plans requiring prior authorization or charging patients significantly more to be on a DOAC. It is conceivable that DOAC underuse may be overrepresented among patients with such coverage precluding their initiation despite longer-term savings in health costs. Therefore, insurers need to better optimize coverage for DOACs to ensure their benefits to our health system may be realized through their wider adoption.

### Limitations

Our study should be interpreted considering the following limitations. First, we could not ascertain the indications for oral anticoagulant use or patient features that may contraindicate DOAC therapy. However as previously stated, most oral anticoagulant use in clinical care is for indications where DOAC use is superior to warfarin. Second, we cannot to determine whether patient preference or economic reasons make patients or prescribers choose warfarin in favor of a DOAC. Economic factors likely play a large role in DOAC uptake at the clinician level. We indirectly addressed whether patient preference accounted for some of the inertia in DOAC uptake and given lower uptake of DOACs among clinicians with stable patient panels, there may be a preference to remain on warfarin, driven by either patients or clinicians. The high use of DOACs among patients newly joining clinician panels suggests that clinicians do consider these drugs more in recent years. However, DOAC use is more convenient for patients, and even at current costs, the lack of need for coagulation monitoring and reduced risk of bleeding events suggest that the drugs are cost-effective at a population level. Therefore, these factors likely do not explain the majority use of warfarin as the preferred anticoagulant among general medical prescribers. Third, we do not include individuals covered through other insurance programs, and therefore, we cannot infer prescriptions patterns among younger, commercially insured patients or those without insurance coverage.

## Conclusions

Among clinicians participating in the Medicare Part D program, there was an increase in DOAC use between 2013 and 2018, but most clinicians continued to use warfarin as their predominant or only anticoagulant instead of DOACs in 2018. These patterns suggest the need to understand the barriers reflected in the clinician inertia with use of DOACs to realize their potential benefits for patients.

## Notes

Supplement. **eFigure 1.** Oral Anticoagulant Beneficiaries by Year **eFigure 2.** Oral Anticoagulant Prescriptions by Year Amongst Additional Specialties **eFigure 3.** Oral Anticoagulant Prescriptions by Graduation Year **eFigure 4**. Beneficiary Categories for Each Provider-Drug Combination Stratified by Year and Specialty
