# Supplementary material for: Clinician Trends in Prescribing Direct Oral Anticoagulants for US Medicare Beneficiaries
Source: JAMA Netw Open. 2021 Dec 6;4(12):e2137288. doi: 10.1001/jamanetworkopen.2021.37288 (PMC8649845; doi:10.1001/jamanetworkopen.2021.37288)
Supplement: Supplement. — eFigure 1. Oral Anticoagulant Beneficiaries by Year eFigure 2. Oral Anticoagulant Prescriptions by Year Amongst Additional Specialties eFigure 3. Oral Anticoagulant Prescriptions by Graduation Year eFigure 4. Beneficiary Categories for Each Provider-Drug Combination Stratified by Year and Specialty [file jamanetwopen-e2137288-s001.pdf]

Supplemental Online Content

Wheelock KM, Ross JS, Murugiah K, Lin Z, Krumholz HM, Khera R. Clinician trends in prescribing direct oral anticoagulants for US Medicare beneficiaries. *JAMA Netw Open*. 2021;4(12):e2137288. doi:10.1001/jamanetworkopen.2021.37288

eFigure 1. Oral Anticoagulant Beneficiaries by Year
eFigure 2. Oral Anticoagulant Prescriptions by Year Amongst Additional Specialties
eFigure 3. Oral Anticoagulant Prescriptions by Graduation Year
eFigure 4. Beneficiary Categories for Each Provider-Drug Combination Stratified by Year and Specialty

This supplemental material has been provided by the authors to give readers additional information about their work.

© 2021 Wheelock KM et al. *JAMA Network Open.*

eFigure 1. Oral Anticoagulant Beneficiaries by Year

Number of beneficiaries prescribed an oral anticoagulant for stratified by year. Beneficiary data are censored if less than 11 beneficiaries receive a given drug. Provider-drug combinations in this category had the number of beneficiaries set to five. In panel A, all beneficiaries included in the study are shown in aggregate. In panels B-E, beneficiary data for cardiologists, internal medicine physicians, family medicine physicians, and advanced practice providers are shown.

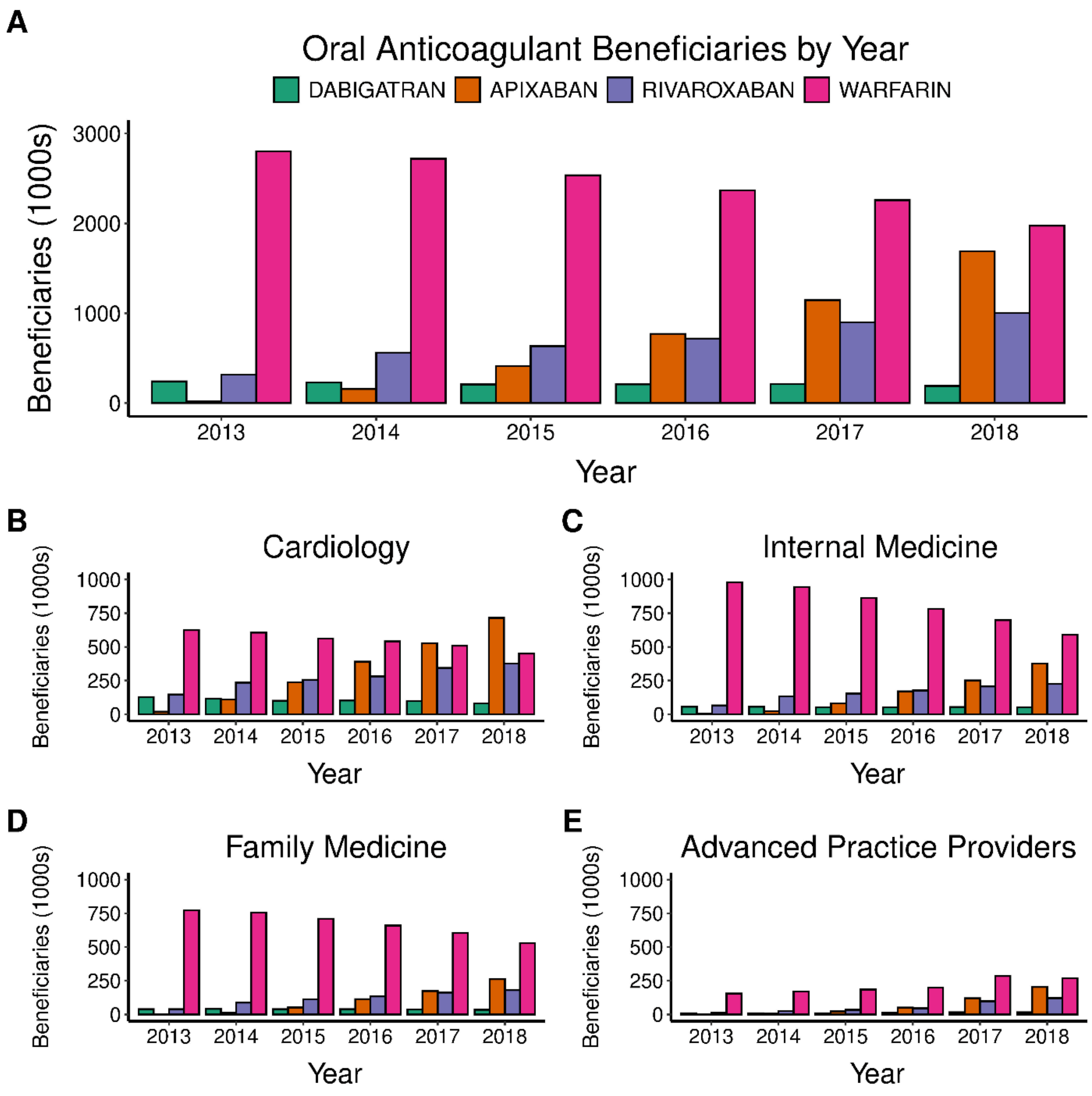

© 2021 Wheelock KM et al. *JAMA Network Open.*

eFigure 2: Oral Anticoagulant Prescriptions by Year Amongst Additional Specialties

Oral anticoagulant claims stratified by year. Panel A includes all claims for hematologist/oncologists, panel B includes claims for all emergency medicine physicians, and panel C includes all claims for vascular surgeons.

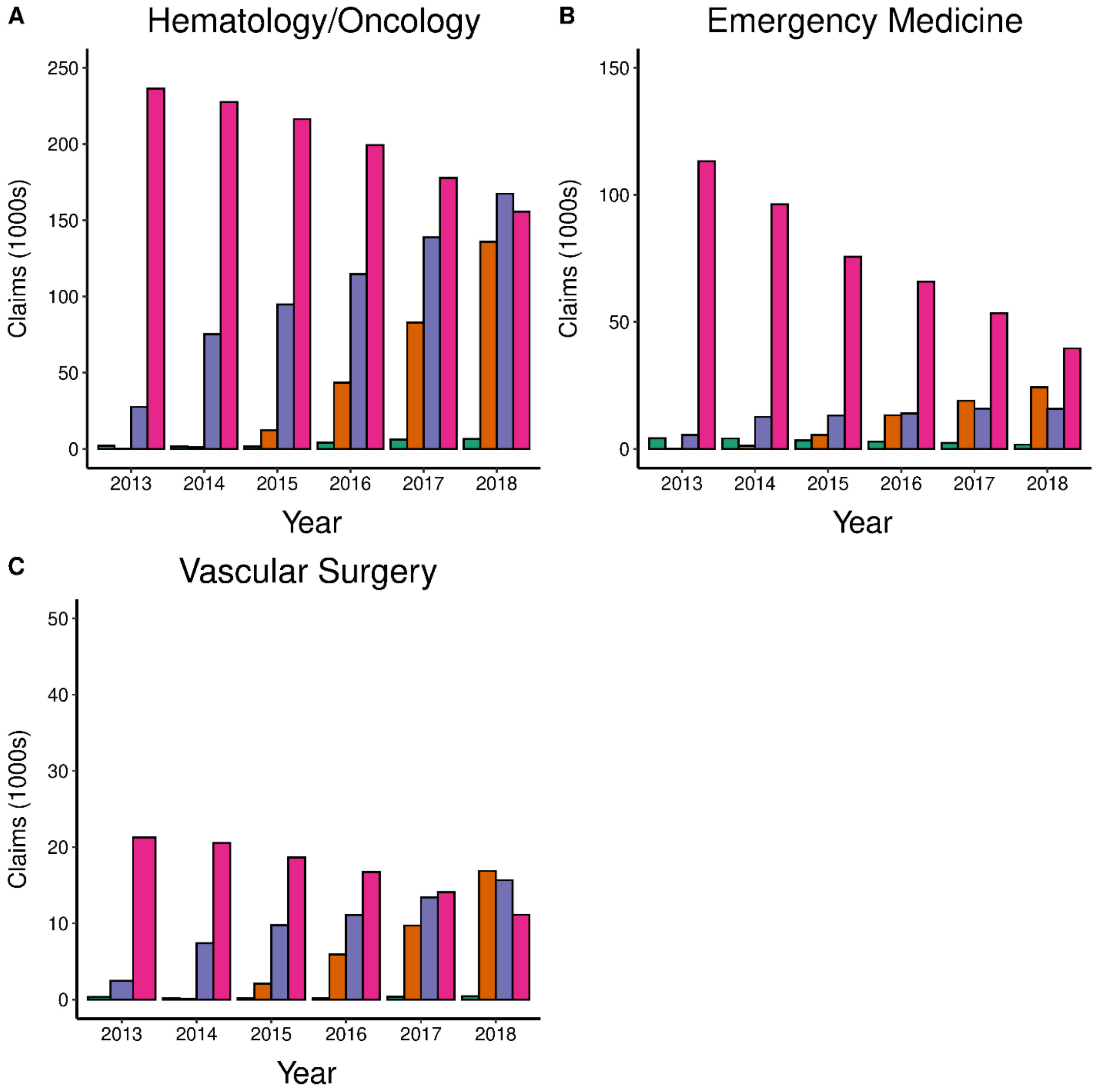

© 2021 Wheelock KM et al. *JAMA Network Open*.

eFigure 3: Oral Anticoagulant Prescriptions by Graduation Year

The proportion of prescriptions for each oral anticoagulant are shown stratified by graduation year.

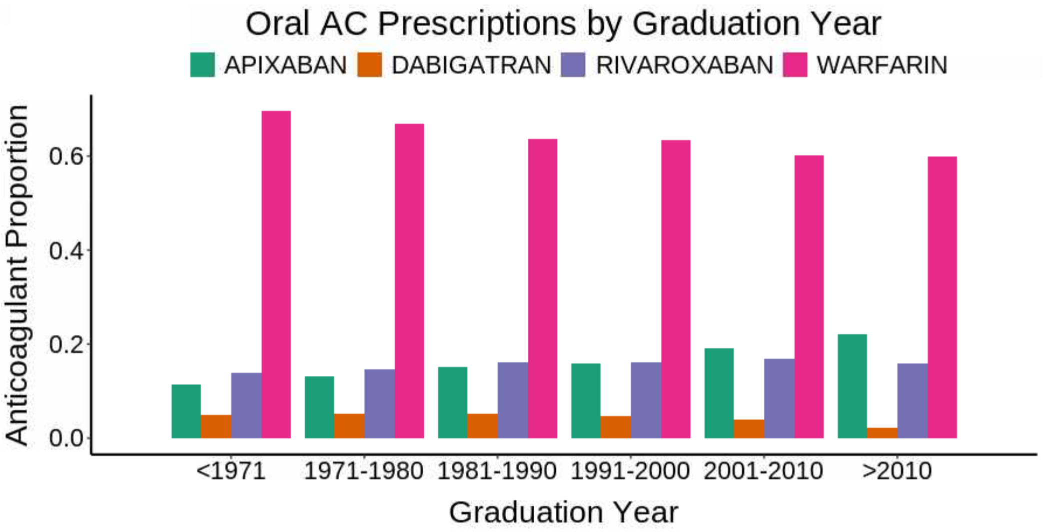

© 2021 Wheelock KM et al. *JAMA Network Open.*

eFigure 4: Beneficiary categories for each provider-drug combination stratified by year and specialty

The number of beneficiaries for each provider-drug combination are shown categorically (<11, 11-20, 21-40, >40 beneficiaries) for each study year. Data were stratified by specialty.

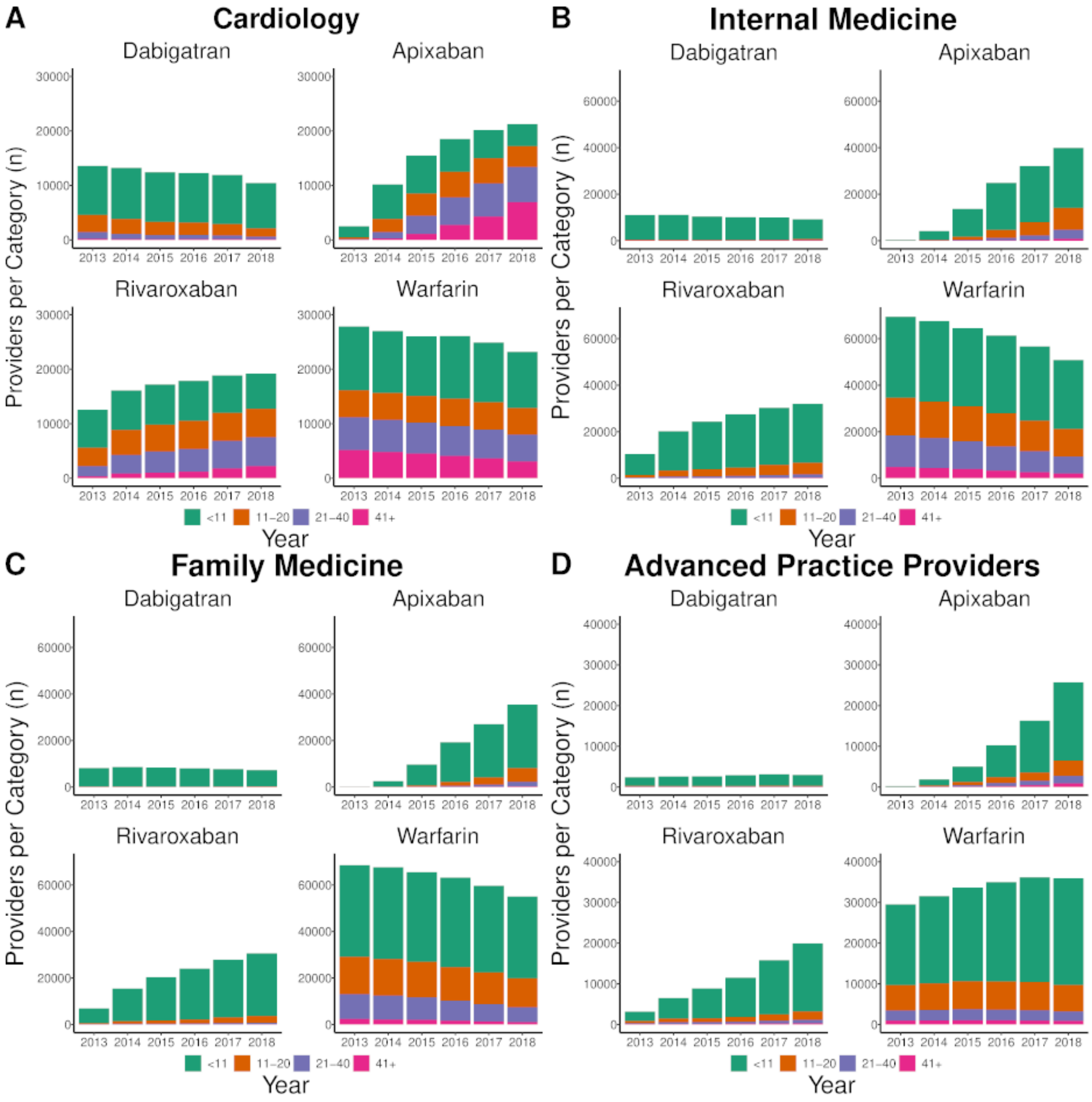

© 2021 Wheelock KM et al. *JAMA Network Open.*
